# Staphylococcus dromedarii sp. nov., isolated from dromedary (Camelus dromedarius)

**DOI:** 10.1099/ijsem.0.007230

**Published:** 2026-07-01

**Authors:** Peter Kuhnert, Isabelle Brodard, Joerg Jores

**Affiliations:** 1 Institute of Veterinary Bacteriology, Vetsuisse Faculty, University of Bern, Bern, Switzerland; 2 International Livestock Research Institute, Nairobi, Kenya

**Keywords:** camel, coagulase-negative, *Staphylococcaceae*, veterinary microbiology

## Abstract

Five bacterial strains of a novel coagulase-negative *Staphylococcus* species were isolated from healthy dromedaries and investigated in a polyphasic taxonomic approach. Phylogenetic analysis based on 16S rRNA gene sequences placed all five strains on a distinct cluster closely related to *Staphylococcus muscae* (99.74%), *Staphylococcus americanisciuri* (99.73%), *Staphylococcus rostri* (99.65%) and *Staphylococcus microti* (99.46%). Whole-genome sequence analysis as well as multilocus sequence analysis confirmed the clustering of the five strains and separated them from the closest related *Staphylococcus* species. Average nucleotide identity was >99% between strains and <85% to any other *Staphylococcus* species, while digital DNA–DNA hybridization values were >95% and <55%, respectively. The DNA G+C content was 36.2–36.4 mol%. Major fatty acids were iso-C_15 : 0_ (61.6%), anteiso-C_15 : 0_ (17.2%) and iso-C_17 : 0_ (6.7%), while the major respiratory quinone was menaquinone-7 (96.7%). The polar lipid profile consisted of diphosphatidylglycerol, phosphatidylglycerol, glycophospholipid, glycolipid, phospholipid and an unknown lipid. The peptidoglycan type was A3*α* l-Lys–Gly_3-4_ (type A11.2). Biochemical markers based on classical as well as commercial identification schemes allowed separation from closely related *Staphylococcus* species. In addition, matrix-assisted laser desorption ionization-time of flight mass spectrometry spectra of the five strains were significantly different from closely related species, allowing rapid identification of the new taxon for which we propose the novel species *Staphylococcus dromedarii* sp. nov. The type strain is IVB6240^T^ (=ILRI248^T^=DSM 115024^T^=CCUG 76602^T^) isolated from the nose of a healthy male dromedary calf in Kenya in 2014.

## Introduction

The genus *Staphylococcus* currently comprises 71 validly published species (https://lpsn.dsmz.de/genus/staphylococcus, accessed on 1 December 2025) and has recently been partially revised [1,3]. Members of the genus are Gram-positive cocci classically arranged in multicellular, grape-like clusters. Species are grouped into coagulase-positive (CoPS) and coagulase-negative (CoNS) staphylococci, whereas most species belong to the latter. While CoPS, like the most prominent *Staphylococcus aureus,* were considered pathogens, the CoNS were mainly regarded as being commensal. This differentiation is becoming less clear-cut, but the separation according to coagulase is still common. Given their generally commensal nature, *Staphylococcus* species are found in a broad range of hosts, including humans, other mammals and birds. While new *Macrococcus* species have been described in camelids, including llamas [4], not much is known about *Staphylococcus* species in camels, and most studies focus on *S. aureus* and mastitis [5]. In a recent study analysing various samples from diseased and apparently healthy dromedaries (*Camelus dromedarius*), we found *S. aureus* to be the predominant staphylococci; however, an additional 10 *Staphylococcus* species could be identified [6]. From that study, five strains isolated in 2014 in Kenya, which were identified as *S. muscae*-like, were further characterized by whole-genome sequencing, phylogenetic analyses, phenotypic and chemotaxonomic investigations. These clearly separated the isolates from known *Staphylococcus* species, and we, therefore, propose a new species *Staphylococcus dromedarii* sp. nov. The five *S. dromedarii* strains were isolated from nasal and eye swabs of healthy animals of both sexes and different ages (Table 1). For detailed characterization, strains were grown from frozen stocks overnight at 37 °C on trypticase soy agar with 5% sheep blood (TSA-SB) (Becton Dickinson).

**Table 1.** *S. dromedarii* strains from dromedary analysed in this study

Strain	Country	Region	Year	Host sex	Host age	Specimen	Status	Genome (bp)	G+C (mol%)	Accession no.
IVB6218	Kenya	Isiolo	2014	Unknown	Calf	Nasal swab	Healthy	2,212,672	36.39	CP094722
IVB6233	Kenya	Laikipia	2014	Female	Adult	Nasal swab	Healthy	2,298,032	36.16	CP094720
IVB6238	Kenya	Laikipia	2014	Female	Adult	Nasal swab	Healthy	2,261,574	36.28	CP094719
IVB6240^T^	Kenya	Laikipia	2014	Male	Calf	Nasal swab	Healthy	2,192,277	36.42	CP094718
IVB6246	Kenya	unknown	2014	Unknown	Unknown	Eye swab	Unknown	2,181,984	36.40	CP094717

## Whole-genome sequences and phylogenetic characterization

**Fig. 1.**
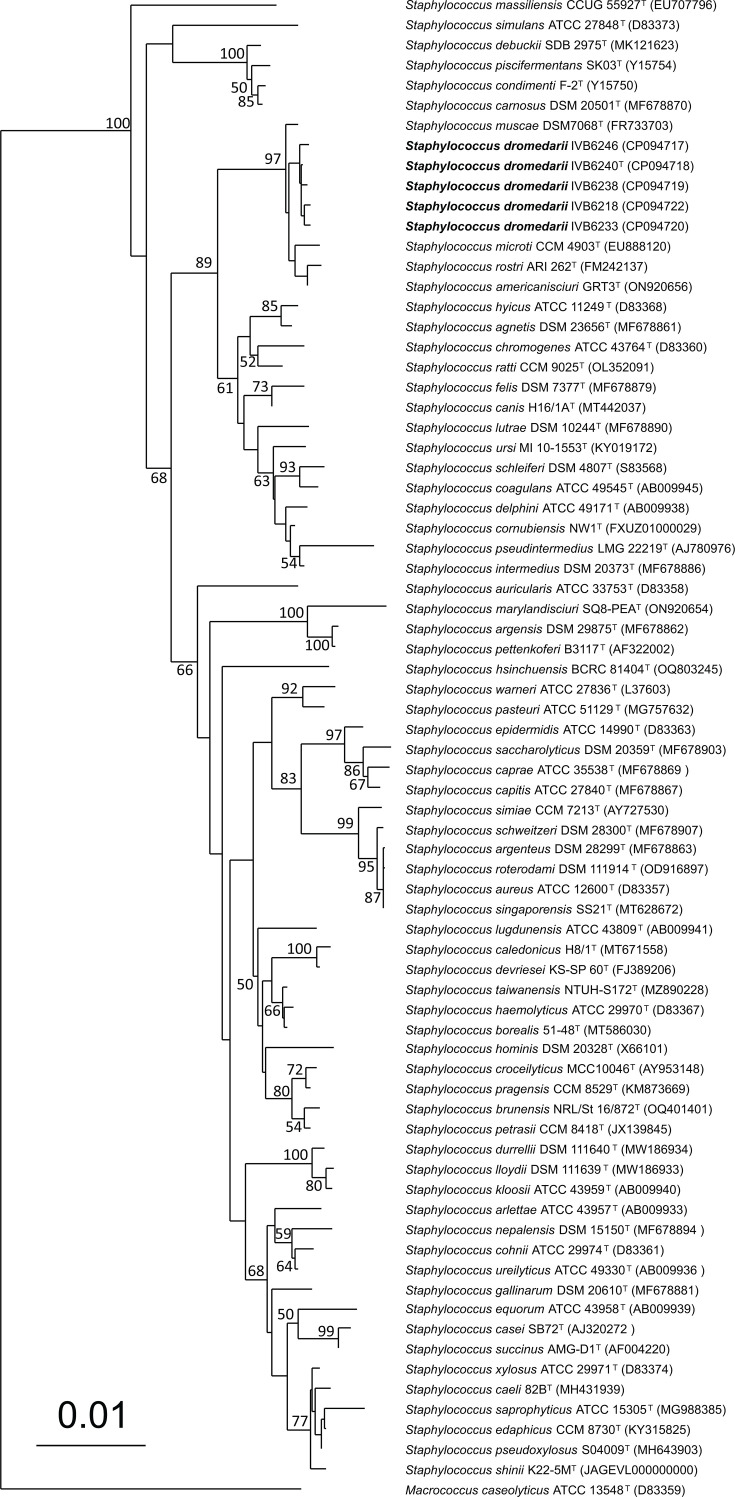
Phylogenetic tree based on 16S rRNA gene sequences of type strains of *Staphylococcus* species and *S. dromedarii* strains. *Macrococcus caseolyticus* was included as an outgroup for rooting the tree that was built in Bionumerics 8.1 using Jukes–Cantor correction and neighbour-joining. Bootstrap values ≥50% based on 500 iterations are given at the branches. The bar represents 1% sequence divergence. Besides the species name, the strain designation and sequence accession number are given.

**Fig. 2.**
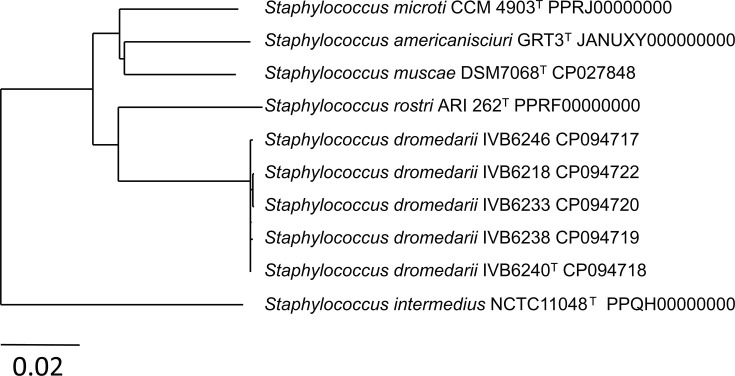
Phylogenetic tree based on multilocus sequence analysis of *S. dromedarii* strains and the closely related *S. americanisciuri*, *S. microti*, *S. muscae* and *S. rostri* type strains. *S. intermedius* type strain was included as an outgroup for rooting the tree. Concatenated gene sequences of *dnaJ*, *gap*, *hsp60*, *rpoB*, *sodA* and *tuf* were used for tree construction in Bionumerics 8.1 with Jukes–Cantor correction and neighbour-joining. The bar represents 2% sequence divergence. Besides the species name, the strain designation and genome sequence accession number are given, from which gene sequences were extracted.

**Fig. 3.**
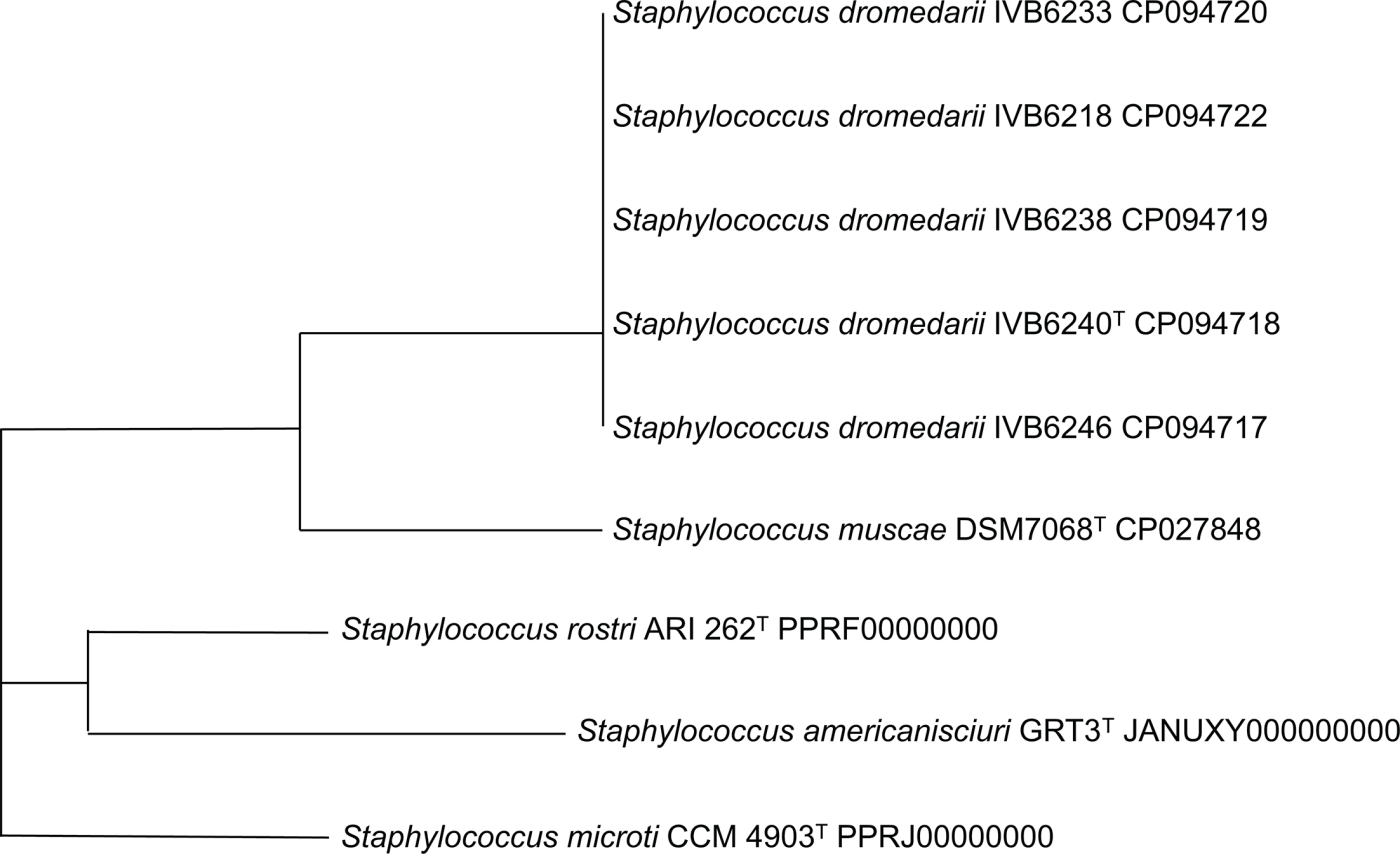
Phylogenetic tree derived from genome sequences of *S. dromedarii* strains and the closely related *S. americanisciuri*, *S. microti*, *S. muscae* and *S. rostri* type strains. A merged tree from the eight trees was built based on individual alignments with each genome sequence as a reference genome using REALPHY 1.13. Besides the species name, the strain designation and genome sequence accession number are given.

Whole-genome sequences (WGS) were generated as described [6] and resulted in complete genomes (Table 1). The genome sizes were about 2.2 Mbp, and the average G+C content was 36.3 mol%. All five *S. dromedarii* strains harbour six rRNA operons. The six 16S rRNA gene copies showed a few ambiguities in all five isolates, which could be confirmed by Sanger sequencing [7]. The corresponding sequence of the type strain has been deposited under accession number PX617434. Phylogenetic analysis based on 16S rRNA gene sequences clustered the five *S. dromedarii* strains on a separate branch within the Hyicus–Intermedius species group [8] close to *Staphylococcus americanisciuri*, *Staphylococcus microti*, S*taphylococcus muscae* and *Staphylococcus rostri* (Fig. 1). To investigate the genetic relationship with these species in more detail, the housekeeping genes *dnaJ*, *gap*, *hsp60*, *rpoB*, *sodA* and *tuf*, which are frequently used for identification of *Staphylococcus* species [9,14], were extracted from the corresponding genome sequences and used for comparison. The phylogenetic analysis based on concatenated genes showed a clear separation of all *S. dromedarii* isolates from the four closest related species (Fig. 2). Similarly, except for *tuf*, gene similarities of the *S. dromedarii* type strain were below the published species cut-off values when compared to the four closest related species and *Staphylococcus intermedius* used as a group representative (Table 2). This was independent of whether the complete gene sequence or only the partial one, as published, was used. In contrast, the 16S rRNA gene sequence similarity included in Table 2 was clearly above the species cut-off value, which is often observed within the genus *Staphylococcus*. However, average nucleotide identity (ANI) values calculated using the OrthoANIu algorithm [15] allowed an unambiguous species separation of the five dromedary isolates with ANI values <85% when compared to the four closest related species (Table 3), which is far below the species cut-off of 95% [16]. Similarly, digital DNA–DNA hybridization (dDDH) values calculated using the Genome-to-Genome Distance Calculator 3.0 [17,18] were <55%, which is clearly below the species cut-off of 70% (Table S1, available in the online Supplementary Material). On the other hand, the *S. dromedarii* isolates showed species identity with ANI values >99% and dDDH values >94%. These findings are corroborated by the WGS analysis using REALPHY [19], which placed all *S. dromedarii* isolates together and separated them from the closest related species (Fig. 3).

**Table 2.** Identities (%) of 16S rRNA, *dnaJ*, *gap*, *hsp60*, *rpoB*, *sodA* and *tuf* genes of *S. dromedarii* type strain IVB6240T with closely related *Staphylococcus* type strains Cut-off values are given based on the indicated publications. For the housekeeping genes, identities based on full-length gene sequence as well as partial sequences from original publications are given, separated by a forward slash.

	Gene (species cut-off values) (reference)						
Type strain	16S rRNA (98.7%) [29]	*dnaJ* (**88.8%) [9]**	*gap* **(96%)** [10]	*hsp60* **(93%)** [14]	*rpoB* **(93.6%)** [11]	*sodA* **(97%)** [12]	*tuf* **(98%)** [13]
*S. muscae* DSM7068T	99.7	84.4/84.1	95.9/95.7	89.6/89.5	93.4/93.3	88.2/87.0	98.8/98.4
*S. microti* DSM22147T	99.5	84.9/83.6	93.4/93.5	87.5/87.1	91.8/93.2	90.0/88.1	98.2/98.7
*S. rostri* ARI262T	99.7	84.2/83.9	93.1/93.1	87.3/86.8	92.4/93.3	90.0/88.4	98.2/98.7
*S. americanisciuri* GRT3T	99.7	85.1/84.6	92.5/92.5	87.6/87.5	90.4/91.4	90.2/90.7	97.6/98.4
*S. intermedius* DSM20373T	98.4	77.8/78.6	85.9/86.8	84.4/85.1	87.4/91.6	81.5/79.5	94.5/95.4

**Table 3.** ANI values between genomes of *S. dromedarii* and closely related *Staphylococcus* species

Strain	IVB6218	IVB6233	IVB6238	IVB6240^T^	IVB6246	ATCC 49910^T^	DSM 21968^T^	DSM 22157^T^
*S. dromedarii* IVB6218 (CP094722)*	100							
*S. dromedarii* IVB6233 (CP094720)	99.89	100						
*S. dromedarii* IVB6238 (CP094719)	99.62	99.59	100					
*S. dromedarii* IVB6240^T^ (CP094718)	99.70	99.74	99.65	100				
*S. dromedarii* IVB6246 (CP094717)	99.49	99.46	99.43	99.48	100			
*S. muscae* ATCC49910^T^ (CP027848)	84.82	84.66	84.82	84.69	84.82	100		
*S. rostri* DSM21968^T^ (PPRF00000000)	80.62	80.60	80.76	80.65	80.84	80.54	100	
*S. microti* DSM22157^T^ (PPRJ00000000)	80.52	80.40	80.42	80.66	80.46	80.47	83.38	100
*S. americanisciuri* GRT3^T^ (JANUXY000000000)	79.73	79.96	79.84	79.79	79.71	79.71	83.76	81.56

*GenBank accession number is given in brackets.

## Phenotypic and chemotaxonomic characterization

Characterization was done in line with Freney *et al*. [20], including more up-to-date methods as well.

All strains were facultative anaerobes, growing well on TSA-SB at 37 °C under aerobic conditions but also under anaerobic conditions in a microincubator (Scholzen). After 48 h aerobic growth, colonies were 1.5–2 mm in diameter, convex, circular, smooth and glossy white. Strains IVB6238, IVB6240^T^ and IVB6246 showed *α*-hemolysis on TSA-SB, while strains IVB6218 and IVB6233 were non-hemolytic. After incubation at 37 °C for 48 h, all strains were non-motile on TSA-SB plates as well as in semi-liquid trypticase soy broth (TSB) (BD BBL™) containing 0.25% agar (BD Difco™ agar). Cells were Gram-stain positive cocci arranged in groups. Catalase activity was observed with all strains when testing interaction with 3% H_2_O_2_ (BD BBL™) on a glass slide. Coagulase was assessed on commercial coagulase test slides (Sigma-Aldrich), with all strains being negative. Oxidase was tested on filter strips using oxidase reagent (BD BBL™), with all strains being negative. All strains were tested on DNase agar (Oxoid) and hydrolysed DNA at 37 °C, but not at 60 °C. Halotolerance was determined by growth at different NaCl concentrations (6.5%, 9% and 12%) in TSB. Thereby, all strains were halotolerant up to 9% NaCl with reduced growth at 12% NaCl. No growth on TSA-SB was observed at 15 °C, but strains IVB6238 and IVB6240^T^ grew at 43 °C, while the growth of IVB6218, IVB6233 and IVB6246 was inhibited at this temperature. Growth in TSB at different pH (pH 4–10 at 0.5 intervals) was tested for the type strain, which grew between pH 6 and 9 with an optimum at pH 7.5.

Extended enzyme profiles were generated on the VITEK2 system (bioMérieux) using GP cards (Table 4). In this system, *S. dromedarii* was negative for d-amygdalin (AMY), phosphatidylinositol phospholipase C (PIPLC), *β*-galactosidase (BGAL), *α*-glucosidase (AGLU), Ala-Phe-Pro arylamidase (APPA), l-aspartate arylamidase (AspA), *α*-mannosidase (AMAN), leucine arylamidase (LeuA), l-proline arylamidase (ProA), *β*-glucuronidase (BGURr), *α*-galactosidase (AGAL), alanine arylamidase (AlaA), tyrosine arylamidase (TyrA), urease (URE), arginine dihydrolase 2 (ADH2s), l-lactate alkalinization (lLATk), d-xylose (dXYL), d-galactose (dGAL), d-ribose (dRIB), lactose (LAC), *N*-acetyl-d-glucosamine (NAG), d-maltose (dMAL), d-mannose (dMAN), d-raffinose (dRAF), pullulan (PUL), cyclodextrin (CDEX), salicin (SAL), polymyxin B resistance (POLYB), bacitracin resistance (BACI) and novobiocin resistance (NOVO). A variable reaction was observed with arginine dihydrolase 1 (ADH1), phosphatase (PHOS), sucrose (SAC), d-trehalose (dTRE) and O/129 resistance (O129R), with the type strain always positive, as well as with l-pyrrolydonyl-arylamidase (PyrA), methyl-B-d-glucopyranoside (MBdG) and d-mannitol (dMAN), with the type strain being negative. Positive reactions were obtained for growth in 6.5% NaCl (NC6.5) and optochin resistance (OPTO).

**Table 4.** Biochemical profiles of strains investigated in this study based on the VITEK 2 GP card 1, IVB6218; 2, IVB6233; 3, IVB6238; 4, IVB6240^T;^ 5, IVB6246; 6, *S. microti* DSM 22147^T;^ 7, *S. rostri* ARI262^T^; 8, *S. muscae* DSM 7068^T^; 9, *S. americanisciuri* GRT3^T^.

	1	2	3	4	5	6	7	8	9
d-Amygdalin	–	–	–	–	–	–	–	–	–
Phosphatidylinositol phospholipase C	–	–	–	–	–	–	–	–	–
d-Xylose	–	–	–	–	–	+	+	–	–
Arginine dihydrolase 1	–	–	–	+	–	+	+	–	+
Beta-galactosidase	–	–	–	–	–	–	–	–	–
Alpha-glucosidase	–	–	–	–	–	–	–	–	–
Ala-Phe-Pro-arylamidase	–	–	–	–	–	–	–	–	–
Cyclodextrin	–	–	–	–	–	–	–	–	–
l-Aspartate arylamidase	–	–	–	–	–	–	–	–	–
Beta-galactopyranosidase	–	–	–	–	–	–	–	–	–
Alpha-mannosidase	–	–	–	–	–	–	–	–	–
Phosphatase	–	–	–	+	–	–	+	–	+
Leucine-arylamidase	–	–	–	–	–	–	–	–	–
l-Proline-arylamidase	–	–	–	–	–	–	–	–	–
Beta-glucuronidase (BGURr)	–	–	–	–	–	–	–	–	–
Alpha-galactosidase	–	–	–	–	–	–	–	–	–
l-pyrrolydonyl-arylamidase	+	+	–	–	–	+	+	+	+
Beta-glucuronidase (BGUR)	–	–	–	–	–	–	–	+	+
Alanine-arylamidase	–	–	–	–	–	–	–	–	–
Tyrosine-arylamidase	–	–	–	–	–	–	–	–	–
d-Sorbitol	–	–	–	–	+	–	–	–	–
Urease	–	–	–	–	–	–	–	–	+
Polymyxin B resistance	–	–	–	–	–	–	–	–	+
d-Galactose	–	–	–	–	–	+	–	–	+
d-Ribose	–	–	–	–	–	–	–	–	–
l-lLactate alkalinization	–	–	–	–	–	–	–	–	–
Lactose	–	–	–	–	–	+	+	–	–
*N*-Acetyl-d-glucosamine	–	–	–	–	–	–	+	+	+
d-maltose	–	–	–	–	–	–	–	–	–
Bacitracin resistance	–	–	–	–	–	–	+	–	–
Novobiocin resistance	–	–	–	–	–	–	–	–	–
Growth in 6.5% NaCl	+	+	+	+	+	+	+	+	+
d-Mannitol	–	+	–	–	+	–	–	–	–
d-Mannose	–	–	–	–	–	–	–	–	–
Methyl-B-d-glucopyranoside	+	+	–	–	–	+	+	+	+
Pullulan	–	–	–	–	–	–	–	–	–
d-raffinose	–	–	–	–	–	–	–	–	–
O/129 resistance	–	–	–	+	+	–	–	–	+
Salicin	–	–	–	–	–	–	–	–	–
Sucrose	+	+	–	+	+	+	+	+	+
d-trehalose	+	+	–	+	+	+	+	+	+
Arginine-dihydrolase 2	–	–	–	–	–	–	–	–	–
Optochinin resistance	+	+	+	+	+	+	+	+	+

Type strains of *S. americanisciuri* GRT3^T^ [21], *S. microti* DSM22147^T^ [22], *S. muscae* DSM7068^T^ [23] and *S. rostri* ARI 262^T^ [24] were included in all comparative assays. *Staphylococcus dromedarii* can be differentiated from the closely related *S. muscae* by *N*-acetylglucosamine and *β*-glucuronidase (BGUR), from *S. microti* by lactose, d-galactose and d-xylose; from *S. rostri* by lactose, d-xylose and *N*-acetylglucosamine; and from *S. americanisciuri* by *β*-glucuronidase (BGUR), urease, d-galactose and *N*-acetylglucosamine reactions, which are all positive for the latter four species.

Analysis of cellular fatty acids, respiratory quinones, polar lipids and peptidoglycan of the *S. dromedarii* type strain was carried out by the Identification Service, Leibniz-Institute DSMZ–Deutsche Sammlung von Mikroorganismen und Zellkulturen GmbH (Braunschweig, Germany). Major fatty acids (>5 %) were iso-C_15 : 0_ (61.6%), anteiso-C_15 : 0_ (17.2%) and iso-C_17 : 0_ (6.7%), allowing further separation of *S. dromedarii* from other species, especially *S. muscae* (Table 5). The major respiratory quinones detected were menaquinones (MK): MK-7 (96.7%), MK-6 (2.6%) and MK-8 (0.7%). The polar lipid profile consisted of diphosphatidylglycerol, phosphatidylglycerol, glycophospholipid, glycolipid, phospholipid and an unknown lipid. The peptidoglycan type was A3*α* l-Lys–Gly_5-6_ corresponding to type A11.2.

**Table 5.** Fatty acid composition of *S. dromedarii* IVB6240T and type strains of *S. microti*, *S. muscae*, *S. rostrii* and *S. americanisciuri*

Fatty acid	*S. dromedarii* IVB6240^T^	*S. microti* DSM22147^T^	*S. muscae* DSM7068^T^	*S. rostri* ARI262^T^	*S. americanisciuri* GRT3^T^
C_10 : 0_	0.2	NR	nr	nr	0.4
iso-C_11 : 0_	1.0	NR	nr	nr	nr
anteiso-C_11 : 0_	0.2	NR	nr	nr	nr
iso-C_13 : 0_	2.8	0.5	1.1	0.7	0.7
iso-C_14 : 0_	–	–	nr	0.4	11.7
C_14 : 0_	1.7	0.6	3.7	1.4	3.9
iso-C_15 : 0_	61.6	51.7	40.9	43.7	9.0
anteiso-C_15 : 0_	17.2	12.6	1.0	19.2	2.4
C_15 : 0_	–	–	nr	0.2	1.3
iso-C_16 : 0_	0.3	0.5	nr	0.7	7.9
C_16 : 0_	3.0	3.7	14.8	6.2	7.7
iso-C_17 : 0_	6.7	13.7	5.8	8.0	0.6
anteiso-C_17 : 0_	2.7	3.9	1.9	5.7	0.3
C_17 : 0_	–	–	nr	0.4	0.9
iso-C_18 : 0_	–	–	nr	0.1	0.4
C_18 : 0_	1.0	4.4	10.7	7.5	6.4
iso-C_19 : 0_	0.5	1.1	1.0	0.8	0.1
anteiso-C_19 : 0_	–	–	nr	0.4	<0.1
C_19 : 0_	–	–	nr	0.4	2.7
C_20 : 0_	0.8	1.2	10.1	4.3	30.0

Data for type strains of *S. microti*, *S. muscae* and *S. rostri* were taken from [24], while data from *S. americanisciuri* were taken from [21]. Values are percentages of total fatty acids. nr, Not reported; –, not detected.

The occurrence of teichoic acid in the novel species was determined by the presence of the TagAHGBD operon in the genome of all five strains. This operon is involved in the cell-wall teichoic acid biosynthesis [25,26].

**Fig. 4.**
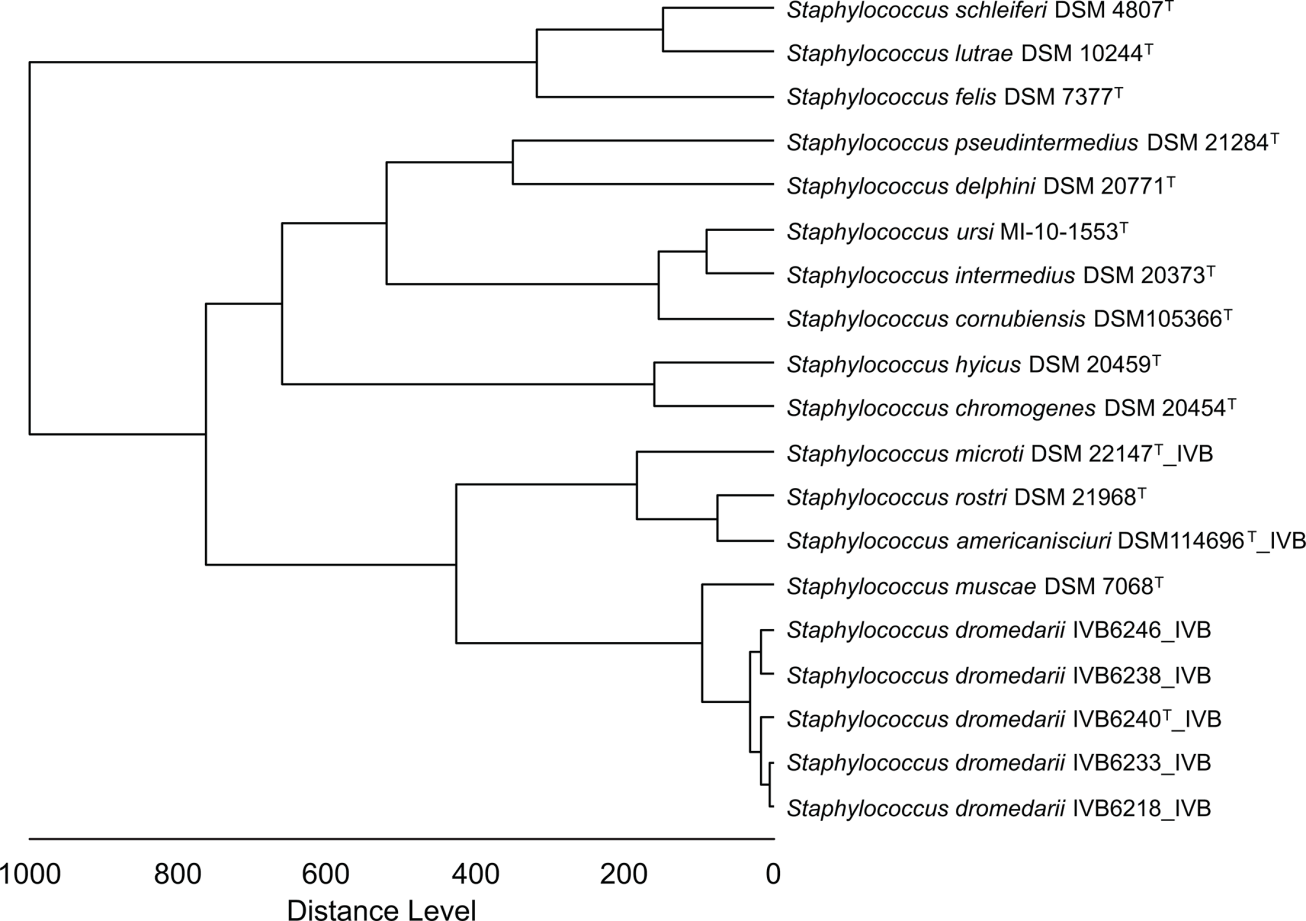
Dendrogram derived from similarity matrices based on newly generated MSP profiles of the *S. dromedarii* strains and *Staphylococcus* species of the Hyicus–Intermedius group contained in the Bruker database. Newly generated MSP profiles are indicated by the suffix ‘IVB’. The distance level is normalized to a maximum value of 1,000.

Matrix-assisted laser desorption ionization-time of flight mass spectrometry (MALDI-TOF MS) main spectra profiles (MSPs) were generated on a Microflex LT (Bruker) from all five isolates grown on TSA-SB at 37 °C. The MSPs were then generated from three to five colonies by the extraction method according to standard procedures [27]. Newly generated spectra and spectra available from the Bruker database were used to create a dendrogram in MALDI Biotyper Compass Explorer 4.1. (Bruker) using standard parameters (Fig. 4). In the MSP-based dendrogram, all *S. dromedarii* isolates cluster together, separated from other closely related species, corroborating its placement as a new species. When identification was done using the standard method, score values were >2.3 with *S. dromedarii* for all five strains, while the score value to the next species (*S. muscae*) was <1.95, making MALDI-TOF MS suitable for diagnostic identification of the new species. The corresponding MSPs of the *S. dromedarii* strains have been deposited (https://maldi-up.ua-bw.de/index_en.asp#EN) and can be shared with interested users [28].

## Description of *Staphylococcus dromedarii* sp. nov.

*Staphylococcus dromedarii* sp. nov. (dro.me.da’ri.i. L. gen. n. *dromedarii*, of a dromedary, a kind of camel).

Cells are Gram-stain positive cocci appearing in groups typical for the genus *Staphylococcus*. Facultative anaerobe growth. After 48 h, colonies on sheep blood agar at 37 °C were 1.5–2 mm in diameter, convex, circular, smooth and glossy white. Some strains, including the type strain, are haemolytic. Catalase reaction is positive, while oxidase and coagulase reactions are negative. Non-motile. Hydrolyses DNA at 37 °C, but not at 60 °C. Halotolerant up to 9% NaCl with reduced growth at 12% NaCl. No growth at 15 °C. Able to grow at up to 43 °C and at pH 6–9.

*S. dromedarii* is negative for d-amygdalin (AMY), phosphatidylinositol phospholipase C (PIPLC), *β*-galactosidase (BGAL), *α*-glucosidase (AGLU), Ala-Phe-Pro arylamidase (APPA), l-aspartate arylamidase (AspA), *α*-mannosidase (AMAN), leucine arylamidase (LeuA), l-proline arylamidase (ProA), *β*-glucuronidase (BGURr), *α*-galactosidase (AGAL), alanine arylamidase (AlaA), tyrosine arylamidase (TyrA), urease (URE), arginine dihydrolase 2 (ADH2s), l-lactate alkalinization (lLATk), d-xylose (dXYL), d-galactose (dGAL), d-ribose (dRIB), lactose (LAC), *N*-acetyl-d-glucosamine (NAG), d-maltose (dMAL), d-mannose (dMAN), d-raffinose (dRAF), pullulan (PUL), cyclodextrin (CDEX), salicin (SAL) and novobiocin resistance (NOVO). A variable reaction is observed with arginine dihydrolase 1 (ADH1), phosphatase (PHOS), sucrose (SAC), d-trehalose (dTRE), O/129 resistance (O129R), l-pyrrolydonyl-arylamidase (PyrA), methyl-B-d-glucopyranoside (MBdG) and d-mannitol (dMAN). Positive reactions are obtained for growth in 6.5% NaCl (NC6.5) and optochin resistance (OPTO).

Major fatty acids are iso-C_15 : 0_, anteiso-C_15 : 0_ and iso-C_17 : 0_. The main menaquinones are MK-7, MK-6 and MK-8. The polar lipid profile consists of diphosphatidylglycerol, phosphatidylglycerol, glycophospholipid, glycolipid, phospholipid and an unknown lipid. The peptidoglycan type of *S. dromedarii* is A3α l-Lys–Gly_5-6_ corresponding to type A11.2.

The average genome size is 2.2 Mbp, and the average G+C content is 36.3 mol% as determined from whole-genome sequences.

The type strain is IVB6240^T^ (=ILRI248^T^=DSM 115024^T^=CCUG 76602^T^) isolated from the nose of a healthy male calf dromedary (*Camelus dromedarius*) in Kenya. The accession number for the 16S rRNA gene sequence of *S. dromedarii* IVB6240^T^ is PX617434. The accession number for the complete genome sequence of strain IVB6240^T^ is CP094718.

## Supplementary material

10.1099/ijsem.0.007230 Table S1.

## Abbreviations

ANI: average nucleotide identity
CoNS: coagulase-negative
CoPS: coagulase-positive
dDDH: digital DNA–DNA hybridization
MALDI-TOF MS: matrix-assisted laser desorption ionization-time of flight mass spectrometry
MK: menaquinone
MSP: main spectra profile
TSA-SB: trypticase soy agar with 5% sheep blood
TSB: trypticase soy broth
WGS: whole-genome sequence
