# Supplementary material for: Staphylococcus dromedarii sp. nov., isolated from dromedary (Camelus dromedarius)
Source: Int J Syst Evol Microbiol. 2026 Jul 1;76(7):007230. doi: 10.1099/ijsem.0.007230 (PMC13322412; doi:10.1099/ijsem.0.007230)
Supplement: Table S1. [file ijsem-76-07230-s001.pdf]

Table S1. dDDH between *Staphylococcus dromedarii* and related *Staphylococcus* species (formula2/formula3)

Strain	IVB6218	IVB6233	IVB6238	IVB6240	IVB6246	ATCC 49910^T	DSM 21968^T	DSM 22157^T
S. dromedarii IVB6218 (CP094722) [#]	100							
S. dromedarii IVB6233 (CP094720)	98.7/99.0	100						
S. dromedarii IVB6238 (CP094719)	96.1/97.8	95.6/96.8	100					
S. dromedarii IVB6240^T (CP094718)	96.5/98.9	96.7/98.5	96.8/98.5	100				
S. dromedarii IVB6246 (CP027848)	95.6/96.6	94.5/95.4	95.0/97.1	95.2/97.5	100			
S. muscae ATCC49910^T (CP027848)	28.8/54.7	28.8/52.8	28.9/53.6	28.9/54.7	28.8/53.8	100		
S. rostri DSM21968^T (PPRF00000000)	24.3/32.4	24.4/32.0	24.2/32.3	24.4/32.1	24.2/32.5	23.9/33.0	100	
S. microti DSM22157^T (PPRJ00000000)	23.8/33.8	23.8/32.9	23.8/33.5	23.8/33.8	23.7/33.8	23.4/35.1	26.9/45.0	
S. americanisciuri GRT3^T (JANUXY000000000)	23.5/27.0	23.5/26.6	23.5/27.1	23.5/27.2	23.5/27.3	23.0/30.9	27.3/44.5	25.1/35.6

[#] GenBank accession number is given in brackets
